# A MIMO Radar-Based DOA Estimation Structure Using Compressive Measurements

**DOI:** 10.3390/s19214706

**Published:** 2019-10-29

**Authors:** Tao Chen, Jian Yang, Muran Guo

**Affiliations:** 1 College of Information and Communication Engineering, Harbin Engineering University, Harbin 150001, China; chentao@hrbeu.edu.cn (T.C.); jyang@hrbeu.edu.cn (J.Y.); 2 Beijing Institute of Remote Sensing Equipment, Beijing 100854, China

**Keywords:** compressive sensing, Cramér–Rao bound, DOA estimation, information theory, MIMO radar

## Abstract

In this paper, we propose a novel direction-of-arrival (DOA) estimation structure based on multiple-input multiple-output (MIMO) radar with colocated antennas, referred to as compressive measurement-based MIMO (CM-MIMO) radar, where the compressive sensing (CS) is employed to reduce the number of channels. Therefore, the system complexity and the computational burden are effectively reduced. It is noted that CS is used after the matched filters and that a measurement matrix with less rows than columns is multiplied with the received signals. As a result, the configurations of the transmit and receive antenna arrays are not affected by the CS and can be determined according to the practical requirements. To study the estimation performance, the Cramér–Rao bound (CRB) with respect to the DOAs of the proposed CM-MIMO radar is analyzed in this paper. The derived CRB expression is also suitable for the conventional MIMO radar by setting the measurement matrix as an identity matrix. Moreover, the CRB expression can work in the under-determined case, since the sum-difference coarray structure is considered. However, the random measurement matrix leads to high information loss, thus compromising the estimation performance. To overcome this problem, we consider that the *a prior* probability distribution of the DOAs associated with the targets can be obtained in many scenarios and an optimization approach for the measurement matrix is proposed in this paper, where the maximum mutual information criterion is adopted. The superiority of the proposed structure is validated by numerical simulations.

## 1. Introduction

Direction-of-arrival (DOA) is an important branch of array signal processing and has gained considerable attention for several decades [1,2]. In conventional DOA estimation problem, the passive (or receive-only) array is used, where the received signals can be either the signals emitted by the targets or the signals that are transmitted by one transmitter and then reflected by the targets. In some literatures, this structure is called single-input multiple-output (SIMO) radar system [3,4]. However, the corresponding estimation performance is limited by the array aperture. The multiple-input multiple-output (MIMO) radar system, which is proposed in Reference [5], has multiple transmit antennas, where multiple independent waveforms are transmitted to detect the targets. By using a bank of matched filters in the receive part, a sum coarray is generated. Thus, the MIMO radar can achieve an extended array aperture, thus improving the estimation accuracy and the number of degrees of freedom (DOFs) [6]. More recently, the massive MIMO concept was introduced in Reference [7]. Then, this concept was extended to large-scale MIMO radar [8], where numerous antennas are used. However, a shortcoming for MIMO radar is that the number of the channels after matched filtering is extremely large. Considering a MIMO radar consisting of *M* transmit antennas and *N* receive antennas, the number of channels is MN. Thus, the system complexity and cost, as well as the computational burden, will dramatically increase when large *M* and *N* are used.

Compressive sensing (CS) is a technique that can recover the sparse original data from a small set of samples [9,10]. The sparse reconstruction method for the DOA estimation problem has been widely used in the passive radar system, for instance, the estimation algorithm [11,12] and the array interpolation [13,14]. We should note that CS is essentially a framework, meaning that, if a problem can be described by a sparse model, then CS can be used to solve the problem, e.g., References [15,16,17]. Recently, due to the superiorities of the MIMO radar system, the applications of CS in the MIMO radar system have been studied in several literatures [18,19,20,21,22,23,24]. In Reference [18], the compressive reconstruction algorithm was extended to the MIMO radar system and a waveform design method was also introduced. In the same proceeding, the CS was employed in a distributed MIMO radar system to reduce the sampling rate [19]. Later, Reference [20] extended the results in Reference [19] into the MIMO radar system with colocated antennas, where the antennas were randomly placed on a disk. In addition, the performance in the presence of a jammer was analyzed in Reference [20]. Then, two approaches were proposed in Reference [21] to optimize the measurement matrix that is used in the CS-based MIMO radar system. In Reference [22], the technique of step frequency was used in the CS-based MIMO radar system. The CS was exploited in the spatial domain to reduce the number of antennas of the MIMO radar system in Reference [23], and the recovery guarantees were also derived. In Reference [24], the performance of several DOA estimation algorithms for the MIMO system is discussed under the practical background of future handsets.

An alternative way to use the CS has been proposed in References [25,26] for the passive radar system, where a measurement matrix is multiplied with the received signal. The number of channels is therefore reduced, thus leading to an improvement on the system complexity and the computational burden. The measurement matrix, denoted as Φ, is essentially the CS kernel, where Φ has less rows than columns and is usually selected in random. Using a random Φ will lead to information loss and is analyzed in Reference [27] through Cramér–Rao bound (CRB). To address this issue, the spatial correlation function and the CRB are used to design the measurement matrix Φ [28]. Furthermore, if the a prior probability density of the incident directions is known, Φ can be optimized iteratively by maximizing the mutual information between Φ and the DOAs [29,30]. The compression structure has also been used in sparse arrays [31,32].

However, existing works on the CS-based MIMO radar mainly focus on reducing the sampling rate or the number of antennas through exploiting the CS, where the sparsity in time domain or spatial domain is considered. As to the research on the use of CS to reduce the number of channels, the background of passive radar is taken into account, while the relevant research under the MIMO radar background is still open. Therefore, in this paper, the CS is employed to reduce the number of channels in an MIMO radar system. It is noted that, different from the spatial compressive MIMO radar proposed in Reference [23], the transmit and receive antenna arrays remain unchanged in the proposed structure. In addition, the transmit or receive antennas are not required to be placed with equal interval. By multiplying the received signal with the measurement matrix, the system complexity as well as the computational burden are effectively reduced. Furthermore, the estimation accuracy is guaranteed due to the large aperture of the transmit and receive antenna array. To study the estimation performance of the proposed structure, the CRB is then explicitly analyzed in this paper. Note that, since the sum-difference coarray structure is exploited, the proposed CRB expression can work in the under-determined scenario. In addition, by setting the measurement matrix Φ as an identity matrix, the CRB expression derived in this paper can be used for the conventional MIMO radar in the under-determined case. To reduce the information loss, we extend the maximum mutual information criterion to the proposed structure. When the prior knowledge of the incident angles is obtained, the estimation accuracy can be improved by using the optimized Φ. Numerical simulations are designed to examine the performance and the CRB analysis of the proposed structure.

This paper is organized as follows. Section 2 introduces the system model and an improved multiple signal classification (MUSIC) algorithm of the proposed CM-MIMO radar. Then, the CRB expression is derived and analyzed in Section 3. The optimization of the measurement matrix is given in Section 4. Numerical simulations are presented in Section 5, and Section 6 concludes this paper.

*Notations*: we use the lowercase letter (e.g., *a*), lowercase bold letter (e.g., a), and uppercase bold letter (e.g., A) to represent the scalars, vectors, and matrices, respectively. The superscripts *, *T*, and *H* denote the complex conjugate, the transpose, and the complex conjugate transpose, respectively. In addition, vec(·) is used to represent the vectorization and expectation operations. The diagonal matrix of which the diagonal entries are given in a is expressed by diag(a). Moreover, tr(A) means the trace of matrix A. The identity matrix with dimension L×L is represented by $I_{L}$. The Kronecker product and Khatri–Rao product are denoted by ⊗ and ∘, respectively.

## 2. The Proposed CM-MIMO Radar Structure

### 2.1. System Model

In this section, we consider a MIMO radar with *M* colocated transmit antennas and *N* colocated receive antennas. Denote two different integer sets as T and R. The locations of the transmit antenna array and the receive antenna array are given as $\left\{{\bar{d}}_{t}d_{0}|{\bar{d}}_{t}\in T\right\}$ and $\left\{{\bar{d}}_{r}d_{0}|{\bar{d}}_{r}\in R\right\}$, respectively, where $d_{0}$ is the half wavelength of the transmitted signals. It is noted that T and R are not required to consists of all continuous integers, indicating that the transmit and receive arrays are not force to be ULAs.

Assume that *Q* targets with DOAs $\left\{\theta_{1},\theta_{2},\cdots ,\theta_{Q}\right\}$ distribute in the far-field area. Since the issue discussed in this paper is DOA estimation, no clutter is considered. The *M* orthogonal waveforms with unit energy are transmitted by the *M* transmit antennas. In addition, omnidirectional antennas are used to transmit and receive signals. Then, after matched filtering, the received signal vector with respect to the *m*th transmit antenna, denoted as $x_{m}\left(t\right)$, is expressed as

(1) $$x_{m}\left(t\right)=\sum_{q=1}^{Q}{\left[a_{t}\left({\bar{\theta }}_{q}\right)\right]}_{m}a_{r}\left({\bar{\theta }}_{q}\right)s_{q}\left(t\right)+n_{m}\left(t\right),$$

where $s_{q}\left(t\right)$ is the reflectivity of the *q*th target with q=1,2,⋯,Q, $n_{m}\left(t\right)$ is the noise, and ${\bar{\theta }}_{q}=d_{0}\sin\theta_{q}/\lambda_{0}$ is the normalized DOA of the *q*th target. It is noted that $s_{q}\left(t\right)$ is assumed to follow complex Gaussian distribution $\mathrm{CN}(0,p_{q})$, where $p_{q}$ is the power of the reflected signal associated with the *q*th target. In addition, we assume that the targets are uncorrelated between each other, indicating that $s_{q}\left(t\right)$ is mutually uncorrelated. Furthermore, $s_{q}\left(t\right)$ is assumed to be independent with the noise. The steering vector of the transmit array and the receive array, i.e., $a_{t}\left({\bar{\theta }}_{q}\right)$ and $a_{r}\left({\bar{\theta }}_{q}\right)$, are respectively defined as

(2) $$a_{t}\left({\bar{\theta }}_{q}\right)={\left[e^{j2\pi {\bar{d}}_{t1}{\bar{\theta }}_{q}},\cdots ,e^{j2\pi {\bar{d}}_{tM}{\bar{\theta }}_{q}}\right]}^{T},$$

(3) $$a_{r}\left({\bar{\theta }}_{q}\right)={\left[e^{j2\pi {\bar{d}}_{r1}{\bar{\theta }}_{q}},\cdots ,e^{j2\pi {\bar{d}}_{rN}{\bar{\theta }}_{q}}\right]}^{T},$$

where ${\bar{d}}_{tm}$ is the position of the *m*th transmit antenna with m=1,2,⋯,M and ${\bar{d}}_{rn}$ is the position of the *n*th receive antenna with n=1,2,⋯,N. As such, assume that no mutual coupling effect is encountered. Then, the receive signal vector x(t) is

(4) x(t)=As(t)+n(t),

where $s\left(t\right)={\left[s_{1}\left(t\right),\cdots ,s_{Q}\left(t\right)\right]}^{T}$ and n(t) is assumed to be the white Gaussian noise with zero mean and the covariance matrix $p_{n}I_{MN}$ in which $p_{n}$ is the noise power. The manifold matrix A is defined as $A=[a\left({\bar{\theta }}_{1}\right),\cdots ,a\left({\bar{\theta }}_{Q}\right)]$, where $a\left({\bar{\theta }}_{q}\right)=a_{t}\left({\bar{\theta }}_{q}\right)⊗a_{r}\left({\bar{\theta }}_{q}\right)$.

**Figure 1:**
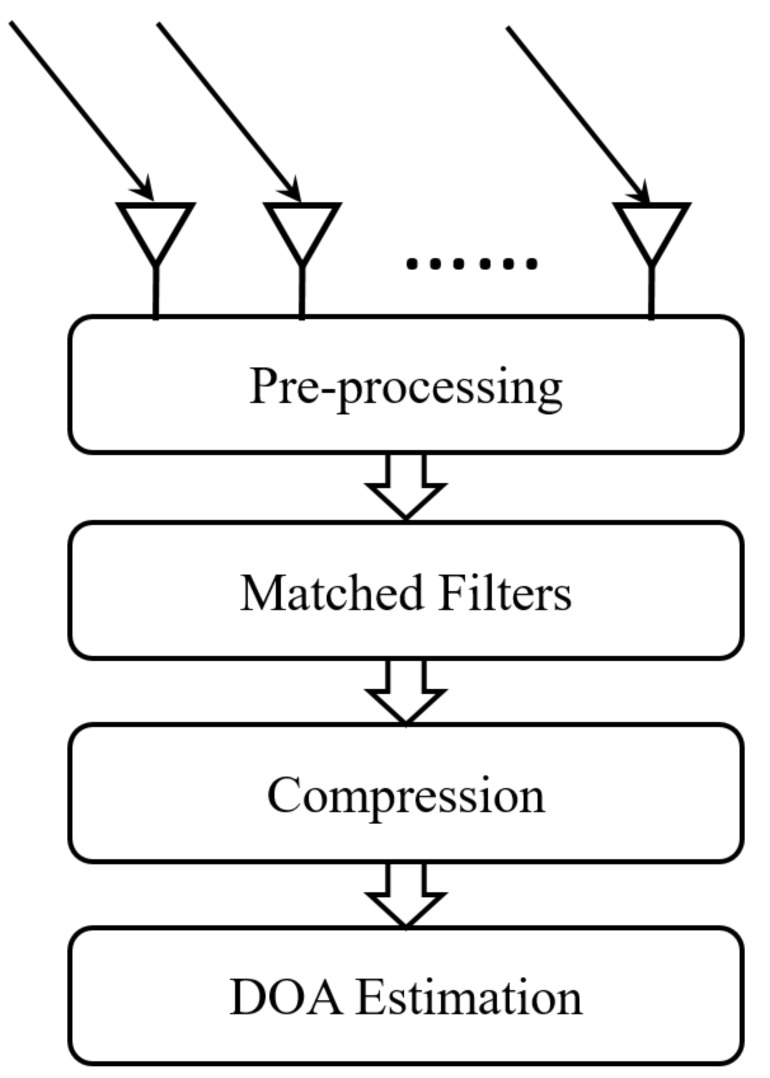
System configuration of the proposed compressive measurement (CM)-multiple-input multiple-output (MIMO) radar structure.

It is found that MN channels are required to process the received data, which leads to a high system complexity. Furthermore, the computational burden is increased due to the high dimension of the received signal. To address this issue, a compression operation is inserted after the matched filters. The compression operation can be described as a complex-valued matrix, denoted as $\Phi \in C^{L\times MN}$ with L<MN as the number of channels after compression. The system configuration is shown in Figure 1. Then, the compressive measurement vector y(t) is

(5) $$y\left(t\right)=\Phi x\left(t\right)=\Phi \left[As(t)+n(t)\right].$$

In order to keep the noise independent between different channels after compression, Φ is assumed to be row orthogonal, i.e., $\Phi \Phi^{H}=I_{L}$. The covariance matrix of y(t) is expressed as

(6) $$R_{\mathrm{yy}}=\Phi AR_{\mathrm{ss}}A^{H}\Phi^{H}+p_{n}I_{L},$$

where $R_{\mathrm{ss}}$ is the covariance matrix of s(t) of which the diagonal elements are the power of the reflected signals, i.e., $R_{\mathrm{ss}}=\mathrm{diag}\left(p\right)$ with $p={\left[p_{1},\cdots ,p_{Q}\right]}^{T}$. In practice, $R_{\mathrm{yy}}$ is approximated by a finite number of signal snapshots. Denote ${\hat{R}}_{\mathrm{yy}}$ as the estimated covariance matrix which is obtained by

(7) $${\hat{R}}_{\mathrm{yy}}=\sum_{t=1}^{T}y\left[t\right]y^{H}\left[t\right],$$

where *T* is the number of snapshots.

### 2.2. Improved MUSIC Algorithm

MUSIC is a high-resolution DOA estimation algorithm and can estimate the DOAs simultaneously. Due to these superiorities, the MUSIC algorithm has gained considerable attention. Several improvements were made on MUSIC to make it available in other scenarios, e.g., sparse array-based DOA estimation [33,34], compressive sensing [35], and radar imaging [36,37,38,39]. Therefore, we develop a MUSIC-based algorithm for the proposed CM-MIMO radar system in this section.

After compression, the conventional MUSIC algorithm fails to estimate the DOAs. A minimum variance distortionless response (MVDR)-based algorithm has been introduced in References [25,29] for the passive radar system, where the compressive measurements are used. The row orthogonality of Φ guarantees that the noise subspace would not spread to the signal subspace. Considering the similarities between MUSIC and MVDR, we introduce an improved MUSIC algorithm here. It is noted that, different with the passive radar system, the steering vector used here is obtained by computing the Kronecker product between the steering vectors of the transmit and receive antenna arrays. The improved MUSIC is also a high-resolution estimation algorithm and can achieve a high estimation accuracy. On the other hand, the number of resolvable targets of the proposed algorithm is limited by the dimension of the covariance matrix, which depends not only on the number of unique lags in the sum coarray but also on the number of channels after compression. We should note that the number of targets *Q* can be pre-estimated using the model order selection technique, e.g., minimum description length (MDL) [40]. Thus, here, we assume that *Q* is already obtained before proceeding the improved MUSIC algorithm. The procedure of the proposed algorithm is briefly summarized as follows.

1. Compute ${\hat{R}}_{\mathrm{yy}}$ using Equation (7);
2. Make the eigen-decomposition for the estimated covariance matrix ${\hat{R}}_{\mathrm{yy}}$;
3. Select the eigenvectors corresponding to the smallest L−Q eigenvalues to obtain the noise subspace, denoted as ${\hat{U}}_{N}$;
4. Devide the spatial domain into a dense grid G, and compute the MUSIC spectrum using the following equation:
  (8) $$P_{\mathrm{MUSIC}}=\frac{1}{a^{H}\left(\theta_{g}\right)\Phi^{H}{\hat{U}}_{N}{\hat{U}}_{N}^{H}\Phi a\left(\theta_{g}\right)},$$
  where g∈G is the index of the spatial grid.
5. Find the largest *Q* peaks of $P_{\mathrm{MUSIC}}$, and the corresponding directions are the estimated DOAs.

## 3. CRB Analysis

As the sparse array concept develops, DOA estimation based on the sparse MIMO radar has been exploited in many literatures, e.g., References [41,42,43]. To make the derived CRB expression available in the under-determined case, the sum-difference coarray structure is involved. Therefore, we first briefly introduce the definitions of the sum coarray and sum-difference coarray.

From the expression of $a({\bar{\theta }}_{q})$, a sum coarray S can be defined as follows:

(9) $$S=\left\{n_{1}+n_{2}|n_{1}\in T,n_{2}\in R\right\}.$$

Note that, for some array structures, there exist overlapping lags in the sum coarray. Let |S| be the cardinality of S; then, a binary matrix, denoted as $J_{s}$, is defined in Definition 1.

**Definition** **1.** *The binary matrix $J_{s}$ is of size MN×|S|, and the column of $J_{s}$ corresponding to the lag m is defined as*

(10) $${\langle J_{s}\rangle }_{:,m}=\mathrm{vec}\left(I(m)\right),\;m\in S,$$

*where I(m) is given as*

(11) $${\langle I\left(m\right)\rangle }_{n_{1},n_{2}}=\left\{\begin{array}{cc} 1 & \mathrm{if}\;n_{1}+n_{2}=m,\\ 0 & \mathrm{otherwise}. \end{array}\right.\;n_{1}\in T,n_{2}\in R.$$

It is noted that, in Definition 1, the triangle bracket ${\langle x_{S}\rangle }_{n}$ represents the value corresponding to the support n∈S. For example, let $x_{S}=\left\{2,3,4\right\}$ and S={−1,0,1}. Then, we have ${\langle x_{S}\rangle }_{-1}=2$, ${\langle x_{S}\rangle }_{0}=3$ and ${\langle x_{S}\rangle }_{1}=4$.

From the sum coarray, it can be found that the aperture corresponding to the MIMO radar is extended, thus improving the number of DOFs and the estimation accuracy. Denote $A_{s}$ as the manifold matrix of the sum coarray. Then, the relationship between A and $A_{s}$ is expressed as

(12) $$A=J_{s}A_{s}.$$

Inspired by the sparse array concept, the sum-difference coarray was exploited to further improve the number of DOFs. The sum-difference coarray is written as

(13) $$D=\left\{n_{1}^{\prime }-n_{2}^{\prime }|n_{1}^{\prime },n_{2}^{\prime }\in S\right\}.$$

Similarly, another binary matrix, denoted as $J_{sd}$, is defined in Definition 2.

**Definition** **2.** *The binary matrix $J_{sd}$ is of size ${\left|S\right|}^{2}\times \left|D\right|$, and the column of $J_{sd}$ corresponding to the lag $m_{d}$ is defined as*

(14) $${\langle J_{sd}\rangle }_{:,m_{d}}=\mathrm{vec}\left(I_{sd}\left(m_{d}\right)\right),\;m_{d}\in D,$$

*where $I_{sd}\left(m_{d}\right)$ is given as*

(15) $${\langle I_{sd}\left(m_{d}\right)\rangle }_{n_{1}^{\prime },n_{2}^{\prime }}=\left\{\begin{array}{cc} 1 & \mathrm{if}\;n_{1}^{\prime }-n_{2}^{\prime }=m_{d},\\ 0 & \mathrm{otherwise}. \end{array}\right.\;n_{1}^{\prime },n_{2}^{\prime }\in S.$$

Denote $A_{sd}$ as the manifold matrix of the sum-difference coarray. The relationship between $A_{sd}$ and $A_{s}$ is expressed as

(16) $$A_{s}^{*}\circ A_{s}=J_{sd}A_{sd}$$

Note that the stochastic model for s(t) is considered in this paper. From Equation (6), it is known that the compressive measurement vector y(t) also follows the complex Gaussian distribution $\mathrm{CN}(0,R_{\mathrm{yy}})$. Define the real-valued parameter vector α as

(17) $$\alpha ={\left[\begin{array}{ccc} {\bar{\theta }}^{T} & p^{T} & p_{n} \end{array}\right]}^{T},$$

where $\bar{\theta }={\left[{\bar{\theta }}_{1},\cdots ,{\bar{\theta }}_{Q}\right]}^{T}$ is the vector of the normalized DOAs. According to Reference [44], the (μ,ν) th entry of the Fisher information matrix (FIM) of the proposed structure is

(18) $${\left[\mathrm{FIM}(\alpha )\right]}_{\mu ,\nu }=T\mathrm{tr}\left(R_{\mathrm{yy}}^{-1}\frac{\partial R_{\mathrm{yy}}}{\partial \alpha_{\mu }}R_{\mathrm{yy}}^{-1}\frac{\partial R_{\mathrm{yy}}}{\partial \alpha_{\nu }}\right).$$

Utilizing the properties of matrix computation [45], Equation (18) can be further simplified as

(19) $$\begin{array}{cc} \; & {\left[\mathrm{FIM}(\alpha )\right]}_{\mu ,\nu }\\ \; & =T{\left[{\left(R_{\mathrm{yy}}^{T}⊗R_{\mathrm{yy}}\right)}^{-\frac{1}{2}}\frac{\partial r_{\mathrm{yy}}}{\partial \alpha_{\mu }}\right]}^{H}\left[{\left(R_{\mathrm{yy}}^{T}⊗R_{\mathrm{yy}}\right)}^{-\frac{1}{2}}\frac{\partial r_{\mathrm{yy}}}{\partial \alpha_{\nu }}\right], \end{array}$$

where $r_{\mathrm{yy}}=\mathrm{vec}\left(R_{\mathrm{yy}}\right)$ is the vectorized covariance matrix. Although the signal power p and the noise power $p_{n}$ are unknown parameters, the normalized DOAs $\bar{\theta }$ are the only parameters to be estimated. Therefore, α can be divided into two parts, and the FIM is then rewritten as

(20) $$\mathrm{FIM}\left(\alpha \right)=T\left[\begin{array}{c} G^{H}\\ \Delta^{H} \end{array}\right]\left[\begin{array}{cc} G & \Delta \end{array}\right],$$

where G and Δ are defined as

(21) $$G={\left(R_{\mathrm{yy}}^{T}⊗R_{\mathrm{yy}}\right)}^{-\frac{1}{2}}\left[\frac{\partial r_{\mathrm{yy}}}{\partial {\bar{\theta }}_{1}},\cdots ,\frac{\partial r_{\mathrm{yy}}}{\partial {\bar{\theta }}_{Q}}\right],$$

(22) $$\Delta ={\left(R_{\mathrm{yy}}^{T}⊗R_{\mathrm{yy}}\right)}^{-\frac{1}{2}}\left[\frac{\partial r_{\mathrm{yy}}}{\partial p_{1}},\cdots ,\frac{\partial r_{\mathrm{yy}}}{\partial p_{Q}},\frac{\partial r_{\mathrm{yy}}}{\partial p_{n}}\right].$$

If FIM is invertible, the CRB expression for $\bar{\theta }$ of the proposed structure is given as

(23) $$\mathrm{CRB}\left(\bar{\theta }\right)=\frac{1}{T}{\left(G^{H}\Pi_{\Delta }^{⊥}G\right)}^{-1},$$

where $\Pi_{\Delta }^{⊥}$ is the null space of Δ, defined as $\Pi_{\Delta }^{⊥}=I-\Delta {\left(\Delta^{H}\Delta \right)}^{-1}\Delta^{H}$.

To derive the CRB expression, the vectorized covariance matrix $r_{\mathrm{yy}}$ must be simplified first. According to the definition of $r_{\mathrm{yy}}$, we have

(24) $$\begin{array}{cc} r_{\mathrm{yy}} & =\mathrm{vec}(R_{\mathrm{yy}})\\ \; & =\sum_{q=1}^{Q}p_{q}\mathrm{vec}\left(\left[\Phi a({\bar{\theta }}_{q})\right]{\left[\Phi a({\bar{\theta }}_{q})\right]}^{H}\right)+p_{n}\mathrm{vec}\left(I_{L}\right)\\ \; & =\left(\Phi^{*}⊗\Phi \right)\sum_{q=1}^{Q}p_{q}a^{*}\left({\bar{\theta }}_{q}\right)⊗a\left({\bar{\theta }}_{q}\right)+p_{n}\mathrm{vec}\left(I_{L}\right). \end{array}$$

By substituting Equation (12) into Equation (24), $r_{\mathrm{yy}}$ can be further simplified as

(25) $$r_{\mathrm{yy}}=\left(\Phi^{*}⊗\Phi \right)\left(J_{s}⊗J_{s}\right)\sum_{q=1}^{Q}p_{q}a_{s}^{*}\left({\bar{\theta }}_{q}\right)⊗a_{s}\left({\bar{\theta }}_{q}\right)+p_{n}\mathrm{vec}\left(I_{L}\right),$$

where $a_{s}\left(\theta_{q}\right)$ is the steering vector of the sum coarray associated with the *q*th target. It is noted that the property (AB)⊗(CD)=(A⊗C)(B⊗D) is exploited in Equation (25). Then, using the equality described in Equation (16), $r_{\mathrm{yy}}$ is rewritten as

(26) $$\begin{array}{cc} r_{\mathrm{yy}} & =\left(\Phi^{*}⊗\Phi \right)\left(J_{s}⊗J_{s}\right)J_{sd}\sum_{q=1}^{Q}p_{q}a_{sd}\left({\bar{\theta }}_{q}\right)+p_{n}\mathrm{vec}\left(I_{L}\right)\\ \; & =FA_{sd}p+p_{n}\mathrm{vec}\left(I_{L}\right), \end{array}$$

where $a_{sd}\left({\bar{\theta }}_{q}\right)$ is the steering vector of the sum-difference coarray associated with the *q*th target and $F=\left(\Phi^{*}⊗\Phi \right)\left(J_{s}⊗J_{s}\right)J_{sd}$.

Thus, the expressions of the partial derivative of $r_{\mathrm{yy}}$ with respect to the normalized DOA, the reflected signal power, and the noise power are

(27) $$\frac{\partial r_{\mathrm{yy}}}{\partial {\bar{\theta }}_{q}}=j2\pi p_{q}F\mathrm{diag}\left(D\right)a_{sd}\left({\bar{\theta }}_{q}\right),$$

(28) $$\frac{\partial r_{\mathrm{yy}}}{\partial p_{q}}=Fa_{sd}\left({\bar{\theta }}_{q}\right),$$

and

(29) $$\frac{\partial r_{\mathrm{yy}}}{\partial p_{n}}=\mathrm{vec}\left(I_{L}\right),$$

respectively. Then, we can obtain G and Δ, expressed as

(30) $$G=j2\pi {\left(R_{\mathrm{yy}}^{T}⊗R_{\mathrm{yy}}\right)}^{-\frac{1}{2}}F\mathrm{diag}\left(D\right)a_{sd}\left({\bar{\theta }}_{q}\right)P,$$

(31) $$\Delta ={\left(R_{\mathrm{yy}}^{T}⊗R_{\mathrm{yy}}\right)}^{-\frac{1}{2}}\left[\begin{array}{cc} Fa_{sd}\left({\bar{\theta }}_{q}\right) & \mathrm{vec}(I_{L}) \end{array}\right],$$

where P=diag(p). As such, the CRB for $\bar{\theta }$ of the proposed structure can be computed using Equations (23), (30) and (31). Since the sum-difference coarray is considered in the CRB expression, we have the following remark.

**Remark** **1.** *The proposed CRB expression also works in the under-determined case, where the number of targets is larger than the number of lags in the sum coarray.*

It is noted that, by setting the measurement matrix as an identity matrix, the proposed DOA estimation structure degenerates to the conventional MIMO radar. On the other hand, the matrix F is simplified as

(32) $$F=\left(J_{s}⊗J_{s}\right)J_{sd}.$$

As this moment, we can obtain the CRB of the conventional sparse MIMO radar by substituting Equation (32) into Equations (30) and (31), which has not been derived before. However, in the under-determined case, the correlation information of the received signal vector must be utilized, which will equivalently generate a group of coherent sources. Then, the improved MUSIC algorithm, demonstrated in Section 2.2, fails to estimate the DOAs. Therefore, the under-determined case is not considered in this paper.

## 4. Optimization of the Measurement Matrix

Denote the a prior probability density function (pdf) of the DOA as f(θ). Then, discretize f(θ) into *K* angular bins, where the length of each bin is represented by Δθ. Denoting $p_{k}^{\prime }=f\left(\theta_{k}\right)\Delta \theta$, we then have $\sum_{k\in K}p_{k}^{\prime }=1$, where K={1,2,⋯,K}. According to the derivations in Reference [29], the mutual information between the compressive measurements and the DOA, denoted as I(y;θ), is approximated as

(33) $$I\left(y;\theta \right)\approx -\ln\sum_{k\in K}p_{k}^{\prime }{\left|R_{\mathrm{yy}|\theta_{k}}\right|}^{-1}-\sum_{k\in K}p_{k}^{\prime }\ln\left|R_{\mathrm{yy}|\theta_{k}}\right|,$$

where $R_{\mathrm{yy}|\theta_{k}}=\Phi \left(p_{s}a\left(\theta \right)a^{H}\left(\theta \right)+p_{n}I_{MN}\right)\Phi^{H}$ with $p_{s}$ representing the reflected source power. Then, the gradient of I(y;θ) with respect to Φ is

(34) $$\nabla_{\Phi }I\left(y;\theta \right)=\frac{\sum_{k\in K}p_{k}^{\prime }{\left|R_{\mathrm{yy}|\theta_{k}}\right|}^{-2}{\left|R_{\mathrm{yy}|\theta_{k}}\right|}^{\prime }}{\sum_{k\in K}p_{k}^{\prime }{\left|R_{\mathrm{yy}|\theta_{k}}\right|}^{-1}}-\sum_{k\in K}p_{k}^{\prime }\frac{{\left|R_{\mathrm{yy}|\theta_{k}}\right|}^{\prime }}{\left|R_{\mathrm{yy}|\theta_{k}}\right|},$$

where ${\left|R_{\mathrm{yy}|\theta_{k}}\right|}^{\prime }$ is the partial derivative of $\left|R_{\mathrm{yy}|\theta_{k}}\right|$ with respect to Φ. For a symmetric matrix A, the following equality holds [46]:

(35) $$\frac{\partial |{\mathrm{BAB}}^{H}|}{\partial B}=2{\left({\mathrm{BAB}}^{H}\right)}^{-1}\mathrm{BA}.$$

Then, ${\left|R_{\mathrm{yy}|\theta_{k}}\right|}^{\prime }$ is expressed as

(36) $${\left|R_{\mathrm{yy}|\theta_{k}}\right|}^{\prime }=2R_{\mathrm{yy}|\theta_{k}}^{-1}\Phi R_{\mathrm{xx}|\theta_{k}},$$

where $R_{\mathrm{xx}|\theta_{k}}=p_{s}a\left(\theta \right)a^{H}\left(\theta \right)+p_{n}I_{MN}$. Substituting Equation (36) into Equation (34), $\nabla_{\Phi }I\left(y;\theta \right)$ can be obtained. Then, the measurement matrix Φ can be optimized iteratively through

(37) $$\Phi^{\left(\xi +1\right)}=\Phi^{\left(\xi \right)}+\kappa \nabla_{\Phi^{\left(\xi \right)}}I\left(y;\theta \right),$$

where ξ represents the ξ th iteration and κ>0 is the step size.

## 5. Simulations

In the following simulations, it is assumed that the normalized DOAs of the targets are ${\bar{\theta }}_{q}=-0.45+0.85\left(q-1\right)/Q$ with q=1,2,⋯,Q. The reflected signals have the same power and are uncorrelated with each other. In addition, the signals are independent from the noise. To evaluate the estimation accuracy, the root mean square error (RMSE) is defined as follows:

(38) $$\mathrm{RMSE}=\sqrt{\frac{1}{IQ}\sum_{i_{0}=1}^{I}\sum_{q=1}^{Q}{\left({\bar{\theta }}_{q}-{\hat{\bar{\theta }}}_{q}^{\left(i_{0}\right)}\right)}^{2}},$$

where ${\hat{\bar{\theta }}}_{q}^{\left(i_{0}\right)}$ is the estimated normalized DOA of the *q* target in the $i_{0}$ th Monte–Carlo trial and *I* is the total number of Monte–Carlo trials. A ULA consisting of 7 antennas is used for both transmitting and receiving, indicating that M=N=7. Thus, for conventional MIMO radar, MN=49 channels are required. We denote this MIMO radar as *MIMO radar 1*, which will be used for comparison in the following simulations. In the proposed structure, the number of channels is compressed to L=25 by using a measurement matrix Φ with dimension 49×25. Each entry in Φ is randomly generated from the standard complex Gaussian distribution CN(0,1). To show the superiority of the proposed structure when the number of channels is fixed, a conventional MIMO radar, denoted as *MIMO radar 2* in the simulations, is considered, where the 5-element ULA is used to transmit and receive signals.

### 5.1. Spatial Spectrum

**Figure 2:**
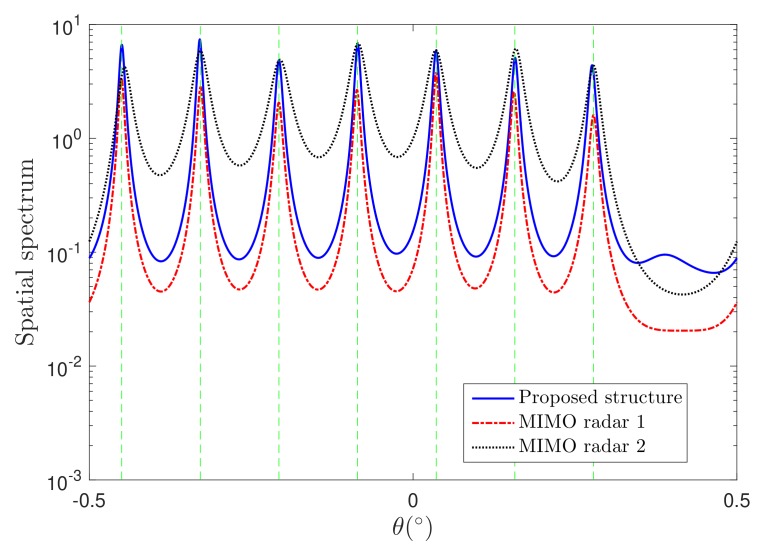
Spatial spectrum comparison.

The performance of the proposed structure is evaluated by comparing the spatial spectrum in the first simulation. It can be found that the number of DOFs for MIMO radar 1 and the proposed structure is 12, while the MIMO radar 2 can resolve 8 targets at most. Hence, we consider Q=7 targets here so that all the structures can estimate the DOAs correctly. Using 0 dB signal-to-noise ratio (SNR) and 100 snapshots, the simulation results are shown in Figure 2. It is observed that the proposed structure obtains a similar spatial spectrum with the MIMO radar 1. On the other hand, the proposed structure can provide a better spectrum than the MIMO radar 2.

### 5.2. RMSE and CRB Simulation

**Figure 3:**
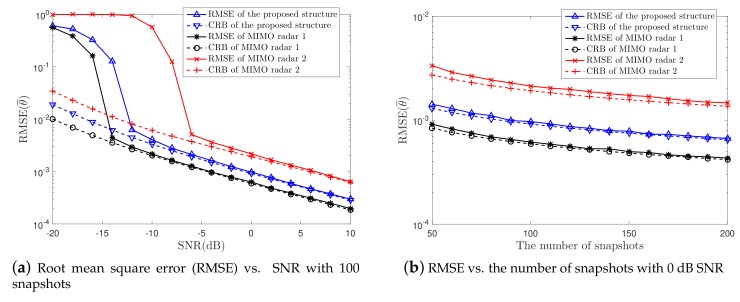
RMSE vs. (**a**) SNR and (**b**) the number of snapshots.

To further assess the estimation accuracy of the proposed structure, the RMSE is computed in this simulation. In addition, the CRB expression derived in this paper is also examined. Similarly, Q=7 targets are considered here and the MIMO radar 1 and MIMO radar 2 are used for comparison. Simulation results are plotted in Figure 3. The number of snapshots is 100 in Figure 3a, and SNR is 0 dB in Figure 3b. Due to the extended array aperture, the proposed structure outperforms the MIMO radar 2, indicating that the proposed structure can achieve a better performance than the conventional MIMO radar when the number of channels is the same. However, the compression operation leads to information lost, which will affect the estimation accuracy, as shown in the simulation results.

In addition, it is observed that the RMSE approaches the CRB as the SNR or the number of snapshots get higher, indicating that the CRB expression derived in this paper can effectively predict the estimation performance of the proposed CM-MIMO radar system and that the improved MUSIC algorithm is the optimal estimation algorithm under the given assumptions. However, we should note that MUSIC cannot always meet the CRB, for instance, in case of the correlated targets or the sparse arrays.

**Figure 4:**
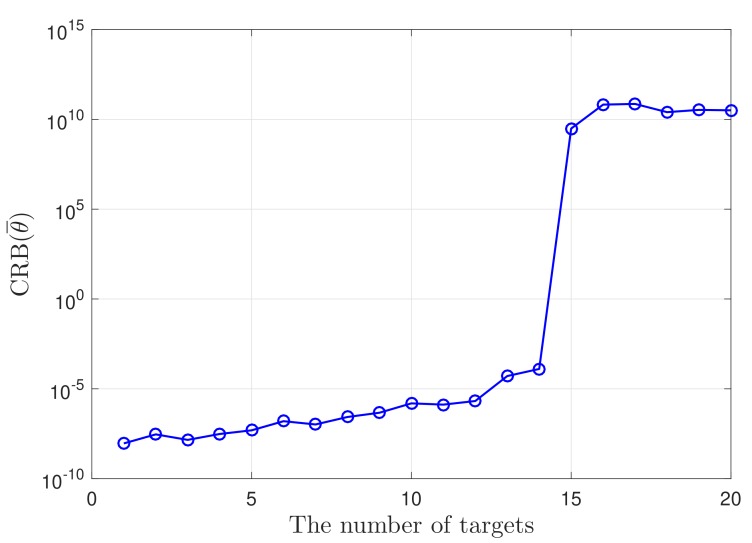
Cramér–Rao bound (CRB) for the normalized direction of arrivals (DOAs) of conventional sparse MIMO radar versus the number of targets.

Furthermore, to verify that the CRB expression also works in the under-determined case for conventional sparse MIMO radar, we consider a coprime MIMO radar, where the antenna positions of the transmit and receive arrays are T={0,3,6,9} and R={0,4,8}, respectively. The number of unique lags in the corresponding sum-difference coarray is 29, meaning that the number of DOFs is 14. Using 0 dB SNR and 100 snapshots, the curve of the CRB versus the number of targets is plotted in Figure 4. It is obvious that the maximum number of resolvable targets indicated by the CRB is also 14, which is consistent with the array configuration.

### 5.3. Optimization of the Measurement Matrix

**Figure 5:**
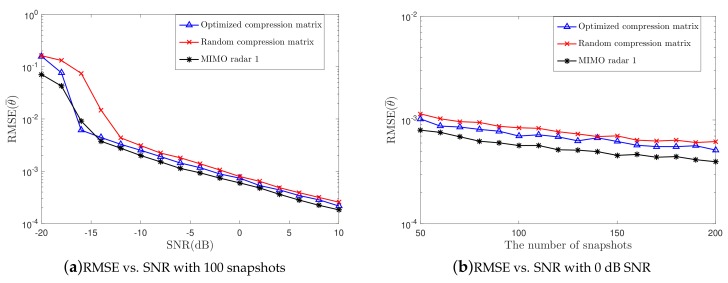
RMSE vs. (**a**) SNR and (**b**) the number of snapshots.

In this section, we assume that there is only one target in the spatial domain and that the incident direction follows the complex Gaussian distribution $\mathrm{CN}(0,5^{2})$. In the optimization procedure, 300 iterations are repeated with κ=0.01 being the step size. It is noted that the exact SNR which is difficult to get is required in the optimization procedure. To address this issue, as analyzed in Reference [47], the optimization is proceeded in high SNR region. The RMSE is computed to evaluate the estimation accuracy, where the proposed structure with random Φ, the proposed structure with optimized Φ, and the MIMO radar 1 are considered. The simulation results are given in Figure 5, where 100 snapshots are used in Figure 5a and 0 dB SNR is used in Figure 5b. As expected, the optimization for the measurement matrix can reduce information loss, thus improving the estimation accuracy, especially in the a low-SNR region. An interesting result is that when, SNR is −16 dB, the proposed structure using optimized Φ can obtain a better performance than the MIMO radar 1. A possible reason is that, in the compression process, most of the useful information remains while the noise is greatly filtered because of the use of optimized Φ. As a result, the SNR is improved after compression. However, as the SNR increases, the information loss is larger than the noise suppression. Thus, MIMO radar 1 still obtains the best estimation accuracy.

## 6. Conclusions

In this paper, we employed the CS with the MIMO radar structure and proposed the CM-MIMO radar system. The proposed structure can effectively reduce the system complexity and computational burden of the estimation algorithm, where the estimation accuracy is also guaranteed. To reveal the performance of the proposed structure, we also derived the corresponding CRB expression, which is suitable for both the traditional case and the under-determined case. Furthermore, an optimization approach based on maximum mutual information criterion was proposed in this paper for the case where the a prior probability density of the targets is known. Simulations examined the performance of the proposed structure and verified the CRB analysis. It is note that the system model constructed in this paper is under the assumption of transmitting narrowband signals. Therefore, developing the system model and the corresponding algorithms that are suitable for the wideband scenario is a direction that is worth exploring in the future.
